# The influence of social physical exercise on the health of middle-aged and older adults: a mediating model of health behavior change mechanisms moderated by age

**DOI:** 10.3389/fpubh.2026.1785261

**Published:** 2026-04-22

**Authors:** Jing Yuan, Gang Mu, Zhaohui Chen

**Affiliations:** 1 Department of Sports, Sichuan University of Media and Communications, Chengdu, China; 2 Department of Public Teaching, Sichuan Railway Vocational College, Chengdu, China

**Keywords:** health behavior change, health status, middle-aged and older, physical exercise, social groups

## Abstract

**Objective:**

To study and discuss the impact of physical exercise on the health status of middle-aged and older adults in the Chinese social environment, with a focus on the mediating role of the health behavior change mechanism and the moderating effect of age.

**Methods:**

This study adopted a cross-sectional design. Established or adapted scales were used to evaluate physical exercise participation, mechanisms of health behavior change, age, and health status. A survey was administered to 473 members of registered sports social organizations in Chengdu, Meishan, Leshan, Nanchong, and Guang’an in Sichuan Province, China. SPSS 27.0 and Amos 21.0 were used to construct a structural equation model to test the hypothesized mediating and moderating effects.

**Results:**

Participation in physical exercise in social groups has a significant direct effect on health status. The indirect effect through the health behavior change mechanism is also statistically significant, supporting the mediation hypothesis. Crucially, these effects are moderated by age, being more pronounced in the older population, reflecting the unique health vulnerability of this group. Control variables (education level, gender, and presence of chronic diseases) do not have a significant impact on the model path.

**Conclusion:**

The health benefits of participating in social group-based physical exercise are primarily driven by the mechanism of individual health behavior change and weaken with age. This study’s findings provide a theoretical and empirical basis for developing age-specific, theory-driven physical exercise intervention measures within social organizations.

## 1 Introduction

The aging global population has significantly strained public health systems, with China’s older population projected to reach 400 million (1). This demographic shift has increased the burden of health issues such as functional decline, chronic disease, cognitive impairment, and depression, which not only diminish quality of life but also demand substantial medical resources (2). In response, China has adopted an “active aging” strategy, emphasizing social participation and physical exercise as key components of health promotion (3). Group-based physical activities (GBPAs), including Tai Chi, square dancing, and sports clubs, have become popular social activities among middle-aged and older individuals (4).

While the health benefits of GBPAs have been established (5, 6) and are attributed to factors such as self-efficacy (7), health responsibility awareness (8), and behavioral intention (9), significant research gaps persist regarding their behavior change mechanisms. First, these mechanisms are often examined individually, with limited analysis of their synergistic effects on multi-dimensional health indicators (4). Second, it is unclear how the mediating effects of these resources evolve with age (10). Third, the unique health behavior change mechanisms associated with culturally specific GBPA forms, such as social sports associations and government-funded social groups, have rarely been explored (6). Consequently, there is an urgent need to develop a new social model to elucidate the relationship among physical exercise, health behavior mechanisms, and overall health status within social groups.

This study employs a moderated mediation model to systematically evaluate the psychosocial effects of physical exercise within social groups. In this model, health behavior change serves as the mediating variable, while age functions as the moderating variable. The findings provide both theoretical and empirical foundations for developing age-specific, culturally appropriate public health interventions that leverage the Chinese GBPA infrastructure.

## 2 Literature review and research hypotheses

### 2.1 Predictive effects of physical exercise on the health of middle-aged and older adults

Research consistently shows that physical exercise serves as a multifaceted predictor of health status in middle-aged and older populations. Extensive studies indicate that adhering to the World Health Organization’s recommended physical activity levels can reduce the risk of heart disease by 20%. This effect is more pronounced for those with uncontrolled blood lipids or lung diseases, suggesting that the dose–response relationship also holds for the Chinese population (11). Among middle-aged and older adults, swimming and dancing have been shown to optimally improve various blood lipid indicators, demonstrating the differential predictive value of exercise types for blood lipid profiles (12). Regarding social-psychological mechanisms, the frequency of sports participation positively predicts group cohesion, and there is a significant chain-mediated effect of social support and psychological capital, with group exercise settings shown to amplify psychological benefits (13). In terms of managing chronic diseases and depression, while depression affects >37% of patients with metabolic syndrome, research demonstrates that physical activity is a reversible protective factor in prediction models (14). Exercise also significantly reduces the probability of depression in disabled populations, further verifying the stability of its predictive ability across different groups (15).

Research indicates that exercises such as Tai Chi, yoga, and health-preserving qigong are effective in reducing pain intensity and enhancing function. While they may not drastically alter quality of life dimensions, they do meet the minimum clinically important difference threshold (16). Additionally, mind–body exercises that aim to manage hypertension have been shown to decrease sympathetic nerve activity, thereby predicting antihypertensive effects (17). Recent studies suggest that blood flow restriction training—particularly low-load, high-pressure modes—holds promise for improving health outcomes in middle-aged and older women (18). Home-based balance training is also notably effective in reducing the likelihood of falls among high-risk individuals, indicating that continuous functional exercise can help maintain mobility (19). In group settings, physical exercise thus offers significant health benefits for middle-aged and older adults, positively affecting cardiovascular, metabolic, psychological, and functional health.

Based on the above, we propose Hypothesis:

*H1*: Engaging in physical exercise within social groups positively influences the health of the older.

### 2.2 Mediating role of the health behavior change mechanism

The impact of changes in health behavior on health outcomes relies on the transformation of individual internal mechanisms. To understand this process, particularly in the context of group physical exercise, it is crucial to dissect the “exercise exposure→health outcome” pathway. A key psychological factor in this mechanism is self-efficacy, namely, an individual’s belief in their ability to perform a specific behavior successfully (20). This is especially pertinent among older adults, for whom higher self-efficacy correlates with health-promoting behaviors (21, 22). Research on chronic diseases indicates that self-care efficacy mediates the relationship between health behaviors and psychological well-being in older patients with hypertension (23). Furthermore, the longer the disease duration, the greater the indirect effect of health literacy on self-management through exercise self-efficacy. This suggests that the “experience–efficacy” spiral in group exercise contexts remains adaptable even in old age (24). Alongside self-efficacy, factors such as social media use (25) and the willingness to share health information (26) play a mediating role in the mechanism of health behavior change. These elements collectively influence how individuals engage in and sustain health-promoting activities.

Cognitive function tends to decline with age, influencing self-efficacy and health behaviors (27). Psychological factors, including self-neglect (28), health responsibility (29), and behavioral intention (30), combined with social and environmental (31) influences, play crucial roles in shaping the health behaviors of older adults (32). Research within group contexts highlights this impact. For instance, Faghih et al. conducted a 6-week intervention using the Health Belief Model (HBM) coupled with social support, and found notable improvements in exercise efficacy and IPAQ scores. This provides causal evidence supporting the “group exercise → self-efficacy → behavioral increment” sequence (33). Similarly, Gao et al. (34) demonstrated that “body image → self-efficacy” mediates the effect of physical activity on overall health, suggesting that self-efficacy remains highly adaptable during the pre-old-age period. Research on health responsibility reveals that social support enhances responsibility attribution, providing a nearly one-third advantage over control groups. This confirms that a sense of health responsibility can be activated within a group context (35), with health awareness acting as a distal antecedent factor (26). Behavioral intention serves as the final proximal link between structural factors and physical activity, with “lack of intention “identified as having the highest attribution risk (36). Furthermore, social ability partially mediates the pathway between physical exercise and mental health, with the correlation strengthening as socioeconomic status declines (10).

We therefore formulate Hypothesis:

*H2*: The mechanisms of health behavior change—namely, self-efficacy, health responsibility, and behavioral intention—mediate the relationship between physical exercise and mental health.

### 2.3 The moderating effect of age

Within the cognitive-behavioral pathway of health behavior change, the moderating variable of age interacts with self-efficacy, health responsibility, and behavioral intention through physiological, psychological, and social aspects, and has an asymmetric moderating effect on the marginal effect of physical exercise, exhibiting a pattern of “first decreasing and then increasing.” As age increases, physical functions begin to decline (37). Research has found that the impact of physical resistance training on lower-limb muscle strength decreases with age, by 3% for every additional year of age (38). As an important psychological factor (39), self-efficacy shows a slope compression after the age of 70. At the same stage, the psychological-cognitive branch is magnified, with the proportion of improvement in psychological resilience driven by perceived health from physical exercise rising to 57% among those aged ≥70. Physical efficacy in the health cognitive channel also becomes the dominant mediator. Through health self-regulation (40), older adults can enhance psychological resilience (41), promote healthy behaviors (42), and build good health literacy (43), thereby forming an effective mechanism for health behavior change.

Community physical exercise can enhance this mechanism. Research demonstrates that prosocial situations strengthen this substitution effect, revealing clear differences in the physical activity levels of the older across age groups (44). The peer chain path becomes ineffective after the age of 65 (45), and peer pressure gives way to prosocial situations. Regarding the residual effects of childhood adversity, negative associations between childhood family risks and current mental health weaken with age until they become insignificant (46), providing a “psychological window” in which physical exercise can activate the health behavior mechanism. Ageing weakens physical self-efficacy, which is amplified by health cognition and prosocial situations in its effect on behavioral intention, resulting in differences in slopes. In conclusion, the following hypothesis is proposed:

*H3*: Age moderates the relationship between physical exercise in social groups and the mechanism of health behavior change.

The impact of age on health status also exhibits the characteristic of “dual thresholds of stage and intensity.” Comprehensive exercise significantly improves the quality of life for the older population, with a steeper slope of net benefits (47). Strenuous-intensity physical activity may be weakly positively correlated with depressive symptoms, and such intense exercise may transform health benefits into costs (48). However, the effect is maintained for moderate-intensity exercise. Physical function declines with age, while mental health scores increase, constituting a phenomenon of “separation of function and perception” (49). Therefore, the following hypothesis is proposed:

*H4*: Age moderates the direct effect of physical exercise in social groups on health status.

### 2.4 Hypotheses and conceptual model

**Figure 1:**
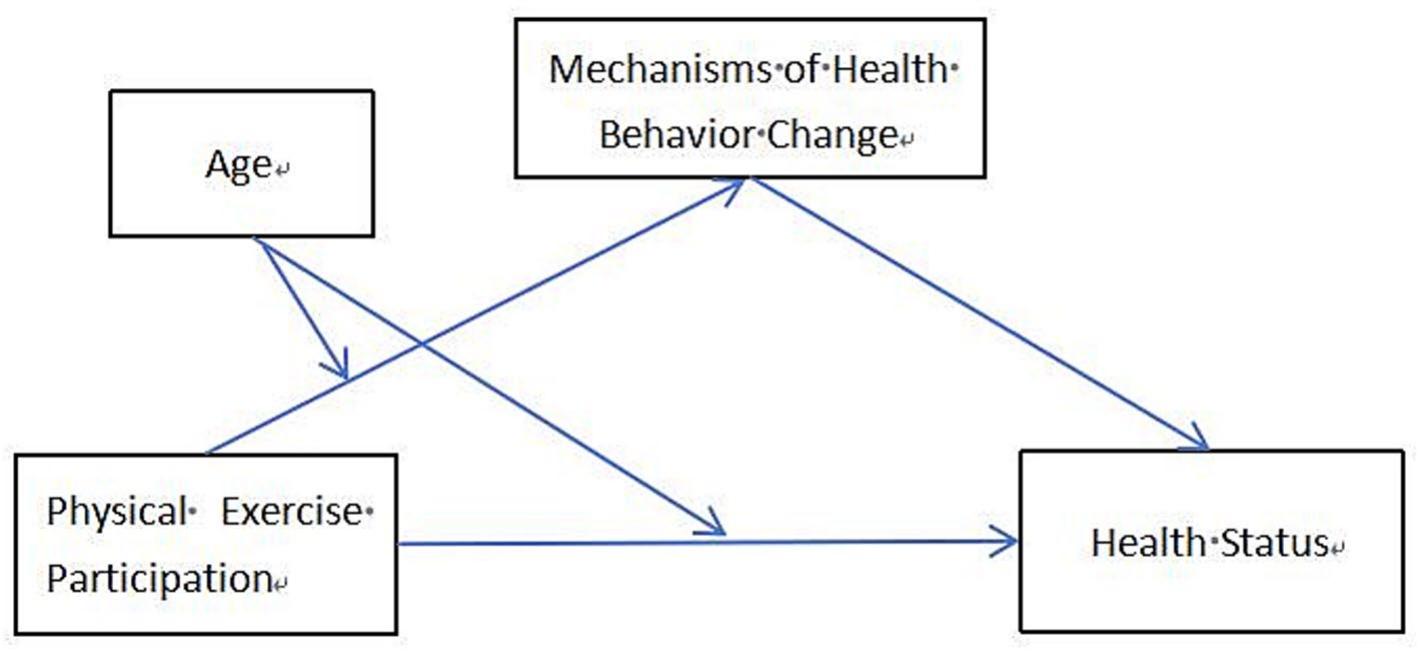
Hypothetical model diagram.

Based on the above literature review and research hypotheses, we constructed a hypothetical model. Figure 1 illustrates the impact of physical exercise participation on health status. The model expresses the direct effects of Hypothesis (H1), the mediating effect of Hypothesis (H2), and the moderating effects of Hypotheses (H3, H4).

## 3 Materials and methods

### 3.1 Participants and procedures

This study employed a cross-sectional questionnaire survey design. Questionnaires were designed using the Wenjuanxing platform and distributed via WeChat between September and October 2025. Data were collected on middle-aged and older adults’ participation levels in group sports activities, the mechanisms of changes in their health behavior, and their health status. All participants signed an electronic informed consent form before completing the questionnaire.

Responses were primarily collected from sports social groups in several cities in the Sichuan region of China, including Chengdu, Meishan, Leshan, Nanchong, and Guang’an. A small-scale pre-test was conducted during the first stage. Based on the results of this pre-test, the questionnaire was revised to ensure respondents could understand and complete it across different cultural contexts, making it suitable for the target group. In the second stage, the organizers of social sports groups and other channels invited participants to complete the questionnaire in large-scale WeChat groups and on social media using relevant QR codes. During this stage, we engaged in real-time communication with the organizers of the social groups to answer any questions that arose during the completion of the questionnaire.

A total of 488 questionnaires were distributed, and 473 valid responses were screened and included in the final analysis, with an effective response rate of 96.9%. Participants had to meet the following inclusion criteria: (1) aged ≥ 30 years; (2) be capable of understanding the questionnaire content and completing it independently; (3) being physically capable of participating in physical exercise (self-assessment); (4) have participated in at least one kind of sports activity organized by social groups (e.g., square dancing, tai chi, power walking, etc.). Participants were excluded if they met the following exclusion criteria: (1) were in a physical condition unsuitable for participating in physical exercise (choosing “completely not allowed” or “not very allowed”); (2) had failed the attention detection question (the final question); (3) spent too little (<2 min) or too much (>25 min) time filling out the questionnaire.

### 3.2 Measurement tools

#### 3.2.1 Physical exercise participation

This adapted the original version of the Physical Activity Scale for the older (PASE) developed by Washburn et al. (50), which has been widely validated (51, 52) and has demonstrated cultural adaptability (53, 54). The scale was simplified for the present study and was semantically fine-tuned by adding the context of “social groups.” The Chinese version of Ainsworth et al.’s (55) scale was used for weighted scoring to evaluate social groups’ physical activity and participation in sports. It comprises five items, including “How often did you participate in the following leisure sports activities in the past week?,” “How long did these activities usually last when you participated in them?,” “What was the intensity of these activities when you participated in them?,” “How persistent were you in participating in the sports activities of the social group?,” and “How many people usually participated in the social group you belong to?” The method for calculating the score is as follows: *Σ* (activity frequency × duration × intensity) + persistence score + group size score. Corresponding weights were assigned as follows: for walking/fitness exercises: frequency (days) × 1.0; for Tai Chi/yoga: frequency(days) × 1.2; for fast walking/square dancing: frequency(days) × 1.3; for running/ball games: frequency(days) × 1.5. Frequency conversion: never = 0 points;1–2 days = 1.5 points; 3–4 days = 3.5 points; 5–6 days = 5.5 points; every day = 7 points. The activity duration ranges from 1 to 5 points, the activity intensity from 1 to 3 points, the participation persistence from 1 to 6 points, and the group size from 1 to 5 points. To facilitate comparisons across studies, the scores for this item were standardized: standardized score = (original score / maximum possible score) × 100. The Cronbach’s alpha of the scale in the present study was 0.711.

#### 3.2.2 Mechanisms of health behavior change

The measure of health behavior change mechanisms comprises three modules: general self-efficacy, health responsibility, and behavioral intention. It includes 10 items from the Chinese revised version of the General Self-Efficacy Scale (GSES) revised by Schwarzer et al. (56), three items on health responsibility from Walker et al.’s scale (57), and two items on behavioral intention based on the work of Ajzen (9). All items were scored on a 5-point Likert scale. The item “I intend to continue participating in social group physical exercises in the next 6 months” in the behavioral intention module, which was scored from 1 (very unlikely) to 5 (very likely), and the other items were scored from 1 (completely inconsistent) to 5 (very consistent). In this study, *α* = 0.947.

#### 3.2.3 Health status

The measure of health status in this study was adapted from the SF-36 Health Survey Short Form. The Chinese standard version (58) of the original (59) comprises 36 questions across eight dimensions. The present study retained 14 questions across three dimensions: physical health, mental health, and functional health. Three self-assessment questions on cognitive function (memory, attention, and complex thinking) were also added. All items were rated on a Likert 5-point scale. The item “How severe is your physical pain?” was reverse-scored. In this study, *α* = 0.949.

### 3.3 Moderating variables

Age was divided into four groups—middle-aged (≤55 years), middle-old (56–65 years), old (66–75 years), and very old (>75 years)—and used as a moderating variable. Since there were only nine people in the very old group (>75), this group was merged with the old group. Finally, three groups were formed and coded: middle-aged = 1, middle-old = 2, old = 3.

### 3.4 Control variables

Gender, educational level, physical condition, participation in community exercise, number of chronic diseases, types of daily medications, and the frequency of medical visits in the past 12 months were used as control variables, as potential confounding factors under consideration.

### 3.5 Data management and statistical analysis

Descriptive statistics, reliability analysis, correlation analysis, KMO value, and Bartlett’s sphericity test were conducted on the questionnaire data using SPSS 27.0. The differences among control variables were explored through an independent-samples T-test and a one-way ANOVA. A structural equation model (SEM) was constructed using Amos 21.0 to test the mediating path of “Physical exercise participation → Health behavior mechanism → Health status” and the moderating effect of “Age.” The mediating effect was tested using bootstrap sampling with 5,000 iterations, and the moderated mediating effect was also examined. The control variables included age, gender, educational level, number of chronic diseases, and types of medications taken. The significance level was set at *p* < 0.05 (two-tailed).

## 4 Research results

### 4.1 Basic information about the samples

Table 1 presents the demographic information of all respondents (*N* = 473). The sample exhibits a significant gender imbalance, with far more females (*n* = 392, 82.9%) than males (*n* = 81, 17.1%). Educational attainment is distributed across all levels, but the proportions vary significantly. Individuals with a high school/secondary vocational school diploma constitute the largest segment (*n* = 153, 32.3%), followed by those with a college diploma (*n* = 133, 28.1%), those with a junior high school diploma (*n* = 116, 24.5%), those with a bachelor’s degree (*n* = 57, 12.1%), and those with a primary school education or below (*n* = 9, 1.9%). The number of respondents with a postgraduate degree or above is the smallest segment (*n* = 5, 1.1%). In terms of physical condition, most respondents reported being able to participate in physical exercise, with 394 answering “yes, completely allowed” (83.3%) and 76 answering “basically allowed, but with restrictions” (16.1%). Only three respondents answered “uncertain” (0.6%). Most respondents regularly participated in physical exercise activities organized by social groups: regularly participate (*n* = 373, 78.9%), occasionally participate (*n* = 81, 17.1%), rarely participate (*n* = 18, 3.8%), never participate (*n* = 1, 0.2%). Regarding chronic diseases and medication use, *n =* 278 had no chronic diseases and took no medication (58.8%), *n* = 159 had chronic diseases and took 1–2 kinds of medication daily (33.6%), *n* = 26 took 3–4 kinds of medication daily (5.5%), *n* = 10 took 5–6 kinds of medication daily (2.1%). Regarding the frequency of seeing a doctor in the past year, respondents answering “rarely (1–2 times)” constituted the largest segment (*n* = 277, 58.6%), followed by those answering “never” (*n* = 45, 9.5%), those answering “sometimes (3–5 times)” (*n* = 113, 23.9%), those answering “often(6–10 times)” (*n* = 26, 5.5%), and those answering “very frequently (more than 10 times)” (*n* = 12, 2.5%).

**Table 1:** Basic information about the respondents.

Variable	Class	Frequency	Percentage (%)
Gender	Male	81	17.1
	Female	392	82.9
Education level	Primary school and below	9	1.9
	junior middle school	116	24.5
	High School/Technical Secondary School	153	32.3
	Junior college	133	28.1
	Undergraduate	57	12.1
	Graduate level or above	5	1.1
Whether your physical condition allows you to participate in physical exercise	Yes, fully allowed	394	83.3
	Allowable, but limited	76	16.1
	indeterminacy	3	0.6
Do you regularly participate in sports activities organized by social groups?	Yes, participate regularly	373	78.9
	Occasional participation	81	17.1
	Rarely attends	18	3.8
	Never participate	1	0.2
Chronic diseases and medication use	No chronic disease, no medication	278	58.8
	Chronic diseases, taking 1–2 medicines a day	159	33.6
	Chronic diseases with daily administration of 3–4 medications	26	5.5
	Chronic diseases, taking 5–6 medications a day	10	2.1
Frequency of medical visits over the past year	never	45	9.5
	Rarely (1–2 times)	277	58.6
	Sometimes (3–5 times)	113	23.9
	Often (6–10 times)	26	5.5
	Very frequent (more than 10 times)	12	2.5

### 4.2 Reliability and validity tests

#### 4.2.1 Reliability test

Reliability is an indicator that assesses the consistency and stability of a measurement tool, reflecting the correlation among items within the tool and the consistency of results across different measurements of the same target. If a questionnaire has high reliability, its data and results are more credible. In this study, reliability was assessed by calculating Cronbach’s alpha for each scale using SPSS 27.0. A *α* coefficient ≥ 0.6 indicates that the questionnaire meets the reliability requirements. The relevant results were obtained by analyzing the measurement scales of each variable.

According to the results presented in Table 2, the Cronbach’s alpha coefficients for the health behavior change mechanism and health status measures are 0.947 and 0.949, respectively, both of which exceed 0.7 and therefore meet the criteria for reliability testing. This indicates that the core scales in this study are all reliable.

**Table 2:** Results of the reliability test.

Variable	Cronbach’s alpha coefficient	Measurement item	Cronbach’s coefficient after item deletion
Mechanisms of health behavior change	0.947	MA1	0.944
		MA2	0.943
		MA3	0.943
		MA4	0.944
		MA5	0.942
		MA6	0.943
		MA7	0.943
		MA8	0.944
		MA9	0.944
		MA10	0.943
		MB1	0.944
		MB2	0.943
		MB3	0.943
		MC1	0.944
		MC2	0.943
Health status	0.949	YA1	0.946
		YA2	0.945
		YA3	0.946
		YA4	0.946
		YA5	0.946
		YB1	0.946
		YB2	0.946
		YB3	0.945
		YB4	0.945
		YB5	0.946
		YB6	0.946
		YB7	0.946
		YC1	0.945
		YC2	0.944
		YD1	0.945
		YD2	0.946
		YD3	0.945

#### 4.2.2 Validity test

Validity is an indicator of whether a questionnaire’s design is reasonable, effective, and accurate. This study used the KMO and Bartlett’s sphericity tests (conducted using SPSS 27.0) to evaluate the questionnaire’s validity. The KMO value ranges between 1 and 0. The closer this value is to zero, the less suitable a questionnaire is for conducting factor analysis; the closer the KMO value is to 1, the higher the degree of correlation between variables, indicating that the questionnaire is more suitable for conducting factor analysis. Relationships between the KMO value and the suitability for factor analysis are categorized as highly suitable (KMO value above 0.8), suitable (0.7–0.8), barely suitable (0.6–0.7), and unsuitable (below 0.5). Bartlett’s sphericity test is used to assess whether the variables are uncorrelated and whether their correlations form an identity matrix. The higher the obtained chi-square value, the more suitable it is for extracting the main factors. The Bartlett’s sphericity test reference Sig is below 0.05, indicating that it meets the standard and has good validity. As shown in Table 3, the KMO values for the health behavior change mechanism and health status are 0.976 and 0.979, respectively, both of which exceed 0.7, indicating suitability for factor analysis. Furthermore, Bartlett’s sphericity test indicates that the significance probability of the chi-square statistic is zero, suggesting that the research scale has good validity.

**Table 3:** KMO and Bartlett’s test.

Variable	KMO sample adequacy measure	Approximate chi-square	Free degree	Conspicuousness
Mechanisms of health behavior change	0.976	4297.056	105	0.00
Health Status	0.979	4656.121	136	0.00

**Figure 2:**
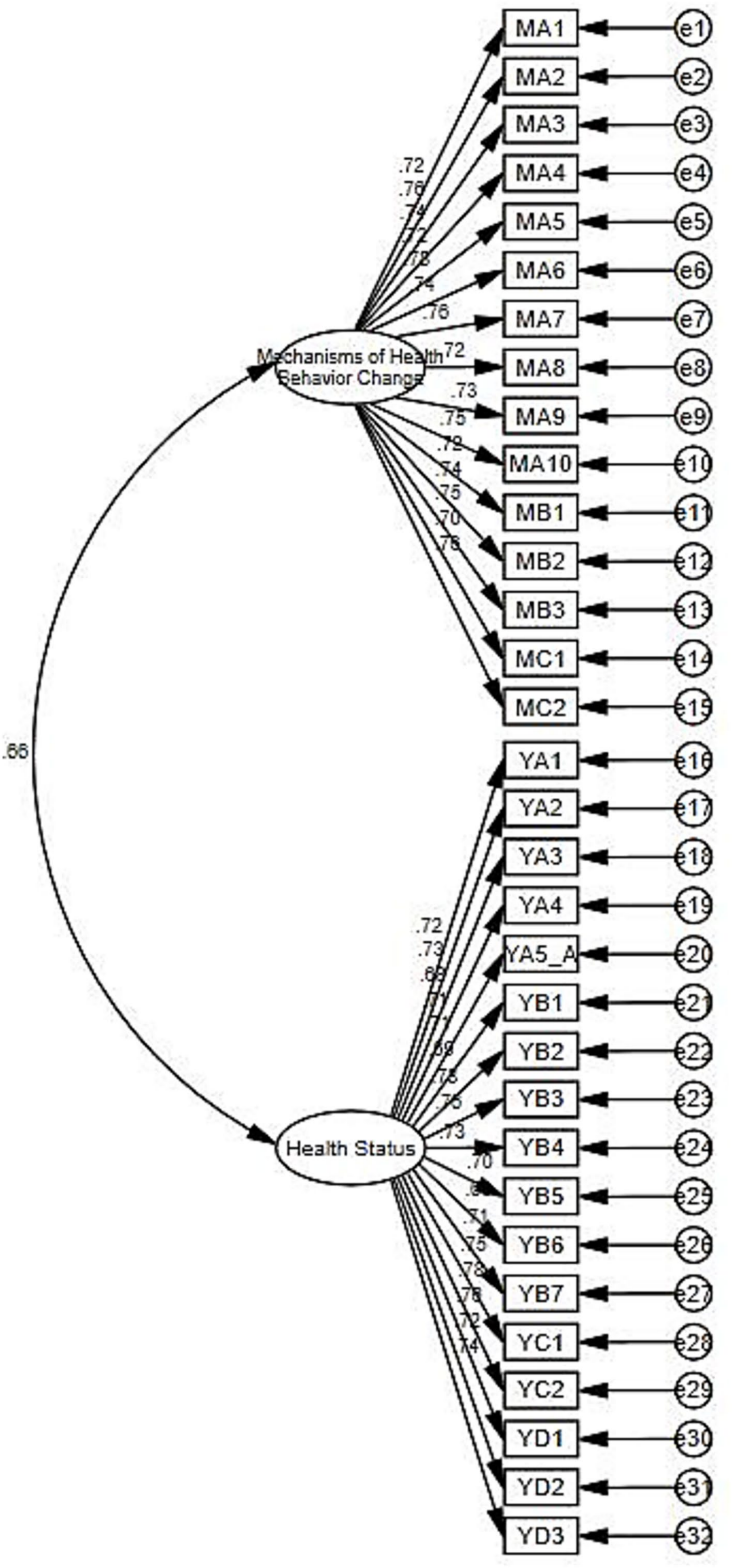
Confirmatory factor analysis (CFA) model diagram.

This study further verified the scale’s convergent and composite validity using AMOS statistical software. As can be seen from the specific results presented in Table 4, the factor loadings for each item on its corresponding latent variable were all greater than 0.4. The AVE values for the core variables were all greater than 0.5, and the CR was greater than 0.7, indicating that the scale’s convergent and composite validity were ideal in this study (see Figure 2).

**Table 4:** Convergent validity and composite validity.

	Unstd	S.E.	C.R.	*p*	Estimate	AVE	CR
MA1 ← Mechanisms of health behavior change	1				0.722	0.5478	0.9478
MA2 ← Mechanisms of health behavior change	1.252	0.076	16.419	***	0.76		
MA3 ← Mechanisms of health behavior change	1.184	0.074	16.081	***	0.745		
MA4 ← Mechanisms of health behavior change	0.994	0.064	15.607	***	0.724		
MA5 ← Mechanisms of health behavior change	1.241	0.074	16.845	***	0.779		
MA6 ← Mechanisms of health behavior change	1.185	0.074	15.946	***	0.739		
MA7 ← Mechanisms of health behavior change	1.107	0.068	16.403	***	0.759		
MA8 ← Mechanisms of health behavior change	0.839	0.054	15.423	***	0.715		
MA9 ← Mechanisms of health behavior change	0.985	0.062	15.853	***	0.735		
MA10 ← Mechanisms of health behavior change	1.23	0.076	16.197	***	0.75		
MB1 ← Mechanisms of health behavior change	1.009	0.065	15.45	***	0.716		
MB2 ← Mechanisms of health behavior change	1.161	0.073	15.972	***	0.74		
MB3 ← Mechanisms of health behavior change	1.046	0.064	16.235	***	0.752		
MC1 ← Mechanisms of health behavior change	1.07	0.071	15.166	***	0.704		
MC2 ← Mechanisms of health behavior change	1.098	0.067	16.379	***	0.758		
YA1 ← Health status	1				0.717	0.5249	0.9494
YA2 ← Health status	0.955	0.061	15.583	***	0.726		
YA3 ← Health status	0.763	0.052	14.635	***	0.683		
YA4 ← Health status	0.886	0.058	15.285	***	0.713		
YA5 ← Health status	0.877	0.058	15.145	***	0.706		
YB1 ← Health status	0.912	0.061	14.867	***	0.694		
YB2 ← Health status	1.109	0.071	15.609	***	0.727		
YB3 ← Health status	1.038	0.064	16.248	***	0.756		
YB4 ← Health status	0.995	0.063	15.737	***	0.733		
YB5 ← Health status	0.867	0.057	15.082	***	0.703		
YB6 ← Health status	0.836	0.056	14.828	***	0.692		
YB7 ← Health status	1.08	0.071	15.313	***	0.714		
YC1 ← Health status	1.105	0.069	16.03	***	0.746		
YC2 ← Health status	1.218	0.073	16.793	***	0.781		
YD1 ← Health status	1.263	0.078	16.246	***	0.756		
YD2 ← Health status	1.169	0.075	15.544	***	0.724		
YD3 ← Health status	1.075	0.068	15.847	***	0.738		

***p < 0.001; AVE, average variance extracted; CR, composite reliability.

### 4.3 Difference analysis

This section examines whether there are significant differences in the core variables across respondent characteristics. Specifically, because gender is a binary variable, an independent-samples t-test was used to assess differences between genders on each variable. Given that educational attainment, physical condition, participation in club exercises, personal chronic disease status, and frequency of doctor visits are all multi-category variables, a one-way analysis of variance (ANOVA) was used to assess whether there are significant differences across these variables.

#### 4.3.1 Gender

Table 5 presents the results of the difference test by gender for health behavior change mechanism, health status, and physical exercise participation. As shown in the table, the F-test statistics for the health behavior change mechanism, health status, and physical exercise participation are 0.463, 1.29, and 1.272, respectively, but there are no significant differences in gender for these variables. Currently, with the popularization of health concepts, men and women have similar cognitions and willingness to change regarding their health behaviors. Additionally, both men and women have equal opportunities for exercise due to the abundance of fitness resources, which could explain why the gender differences are not significant in terms of health behavior change mechanism, health status, and physical exercise participation.

**Table 5:** Analysis of gender differences.

Variable	Gender	*N*	Average value	Standard error	*F*	Conspicuousness
Mechanisms of health behavior change	Male	81	3.763	0.604	0.463	0.497
	Female	392	3.692	0.627		
Health status	Male	81	3.739	0.589	1.29	0.257
	Female	392	3.748	0.533		
Physical exercise participation	Male	81	140.294	82.774	1.272	0.26
	Female	392	127.522	77.186		

#### 4.3.2 Education level

Table 6 presents the results of the tests examining differences in health behavior change mechanisms, health status, and physical exercise participation across education levels. As shown in the table, the F-test statistics for the health behavior change mechanism, health status, and physical exercise participation are 0.566, 0.689, and 1.038, respectively; however, no significant differences were found across different education levels. This could be explained by the highly developed modern network, which allows people with varying levels of education to conveniently access health information and exercise guidance. Additionally, as overall social health promotion efforts continue to increase, people generally attach greater importance to health.

**Table 6:** Analysis of differences in educational attainment.

Variable	Class	*N*	Average value	Standard error	*F*	Conspicuousness
Mechanisms of health behavior change	Primary school and below	9	3.504	0.748	0.566	0.726
	Junior middle school	116	3.732	0.684		
	High School/Technical Secondary School	153	3.663	0.609		
	Junior college	133	3.754	0.607		
	Undergraduate	57	3.673	0.567		
	Graduate level or above	5	3.693	0.441		
Health status	Primary school and below	9	3.506	0.586	0.689	0.632
	Junior middle school	116	3.716	0.592		
	High School/Technical Secondary School	153	3.781	0.526		
	Junior college	133	3.770	0.533		
	Undergraduate	57	3.707	0.506		
	Graduate level or above	5	3.656	0.508		
Physical exercise participation	Primary school and below	9	114.678	114.445	1.038	0.394
	Junior middle school	116	134.901	86.636		
	High School/Technical Secondary School	153	129.735	73.298		
	Junior college	133	133.094	78.071		
	Undergraduate	57	118.865	68.726		
	Graduate level or above	5	69.100	28.055		

#### 4.3.3 Physical condition

Table 7 presents the results of difference tests for the health behavior change mechanism, health status, and physical exercise participation by physical condition. The F-test statistics for the health behavior change mechanism, health status, and physical exercise participation are 4.808, 7.953, and 7.131, respectively, all with significance levels below 0.01, indicating that these differences are statistically significant at the 1% level. Individuals in better physical condition have greater physical fitness and are more willing and able to participate in physical exercise. However, those in poor physical condition are limited by their lack of physical fitness, which reduces their enthusiasm for exercise, thereby showing significant differences across the three variables.

**Table 7:** Analysis of differences in physical conditions.

Variable	Class	*N*	Average value	Standard error	*F*	Conspicuousness
Mechanisms of health behavior change	Yes, fully allowed	394	3.743	0.621	4.808	0.009
	Allowable, but limited	76	3.509	0.599		
	indeterminacy	3	3.467	0.702		
Health status	Yes, fully allowed	394	3.790	0.547	7.953	0.000
	Allowable, but limited	76	3.523	0.454		
	indeterminacy	3	3.759	0.790		
Physical exercise participation	Yes, fully allowed	394	135.612	79.056	7.131	0.001
	Allowable, but limited	76	99.070	65.614		
	Indeterminacy	3	130.667	110.328		

#### 4.3.4 Participation in club exercises

Table 8 presents the results of the difference tests for the health behavior change mechanism, health status, and physical exercise participation with respect to club exercise participation. The F-test statistics of the health behavior change mechanism, health status, and physical exercise participation are 0.658, 0.19, and 6.786, respectively. Differences in physical exercise participation are significant at the 1% level, but no significant differences are observed for the health behavior change mechanism or health status according to various club exercise participation situations. Clubs can provide exercise guidance, thereby stimulating individuals’ motivation to exercise, which could explain the significant difference in physical exercise participation across different club exercise participation situations.

**Table 8:** Differential analysis of participation in community exercises.

Variable	Class	N	Average value	Standard error	*F*	Conspicuousness
Mechanisms of health behavior change	Yes, participate regularly	373	3.715	0.610	0.658	0.578
	Occasional participation	81	3.696	0.689		
	Rarely attends	18	3.507	0.608		
	Never participate	1	3.867			
Health status	Yes, participate regularly	373	3.750	0.545	0.19	0.903
	Occasional participation	81	3.749	0.559		
	Rarely attends	18	3.657	0.435		
	Never participate	1	3.889			
Physical exercise participation	Yes, participate regularly	373	137.792	79.235	6.786	0.00
	Occasional participation	81	101.355	68.629		
	Rarely attends	18	88.694	57.423		
	Never participate	1	149.800			

#### 4.3.5 Chronic disease status

Table 9 presents the results of the difference tests for the health behavior change mechanism, health status, and physical exercise participation in relation to respondents’ reported chronic disease conditions. The F-test statistics for the health behavior change mechanism, health status, and physical exercise participation are 0.672, 0.161, and 1.854, respectively; however, no significant differences were observed across these variables by chronic disease status. Although patients with chronic diseases are affected by their conditions, modern medical technology can effectively manage them. Additionally, to improve their health, patients with chronic diseases will choose appropriate exercise methods based on their conditions and have the same exercise needs and actions as healthy people.

**Table 9:** Differential analysis of self-reported chronic diseases.

Variable	Class	*N*	Average value	Standard error	*F*	Conspicuousness
Mechanisms of health behavior change	No chronic disease, no medication	278	3.735	0.622	0.672	0.569
	Chronic diseases, taking 1–2 medicines a day	159	3.663	0.636		
	Chronic diseases with daily administration of 3–4 medications	26	3.674	0.552		
	Chronic diseases, taking 5–6 medications a day	10	3.553	0.655		
Health status	No chronic disease, no medication	278	3.753	0.570	0.161	0.923
	Chronic diseases, taking 1–2 medicines a day	159	3.743	0.505		
	Chronic diseases with daily administration of 3–4 medications	26	3.746	0.480		
	Chronic diseases, taking 5–6 medications a day	10	3.633	0.556		
Physical exercise participation	No chronic disease, no medication	278	130.794	80.341	1.854	0.137
	Chronic diseases, taking 1–2 medicines a day	159	128.471	73.637		
	Chronic diseases with daily administration of 3–4 medications	26	145.567	82.377		
	Chronic diseases, taking 5–6 medications a day	10	77.990	66.963		

#### 4.3.6 Frequency of doctor visits

Table 10 presents the results of the difference tests for the health behavior change mechanism, health status, and physical exercise participation in relation to the frequency of doctor visits. The F-test statistics for the health behavior change mechanism, health status, and physical exercise participation are 0.135, 0.708, and 0.367, respectively; however, no significant differences are observed in these variables across respondents with different doctor-visit frequencies. Regardless of the frequency with which patients visit the doctor, all generally recognize the importance of health, which may explain why there are no significant differences in the health behavior change mechanism, health status, and physical exercise participation across respondents with different doctor-visit frequencies.

**Table 10:** Analysis of the differences in the frequency doctor visits.

Variable	Class	*N*	Average value	Standard error	*F*	Conspicuousness
Mechanisms of health behavior change	Never	45	3.711	0.632	0.135	0.969
	Rarely (1–2 times)	277	3.705	0.621		
	Sometimes (3–5 times)	113	3.679	0.642		
	Often (6–10 times)	26	3.744	0.589		
	Very frequent (more than 10 times)	12	3.794	0.626		
Health status	Never	45	3.693	0.583	0.708	0.587
	Rarely (1–2 times)	277	3.756	0.548		
	Sometimes (3–5 times)	113	3.741	0.532		
	Often (6–10 times)	26	3.673	0.499		
	Very frequent (more than 10 times)	12	3.958	0.480		
Physical exercise participation	Never	45	130.149	81.494	0.367	0.832
	Rarely (1–2 times)	277	132.034	79.080		
	Never	113	122.492	75.869		
	Rarely (1–2 times)	26	130.142	69.791		
	Sometimes (3–5 times)	12	141.417	92.836		

### 4.4 Correlation analysis

Pearson correlation analysis was employed to investigate the correlations among the study’s core variables (health status, physical exercise participation, health behavior change mechanism, and age). The correlation coefficient measures the degree of correlation between variables and ranges from −1 to 1, with values closer to either end indicating a stronger correlation. The results are presented in Table 11.

**Table 11:** Results of correlation analysis.

	Average value	Standard error	Health Status	Physical exercise participation	Mechanisms of health behavior change	Age
Health status	3.747	0.543	–			
Physical exercise participation	129.709	78.229	0.368***	–		
Mechanisms of health behavior change	3.704	0.623	0.628***	0.320***	–	
Age	2.070	0.678	−0.416***	0.156***	−0.279***	–

*p < 0.05; **p < 0.01; ***p < 0.001.

All correlations are significant at the 1% level. Physical exercise participation is significantly positively correlated with health status (*r* = 0.368, *p* < 0.01). Similarly, the health behavior change mechanism and health status are positively correlated (*r* = 0.628, *p* < 0.01). Age is significantly negatively correlated with health status (*r* = *−*0.416, *p <* 0.01). Physical exercise participation and the health behavior change mechanism are significantly positively correlated (*r* = 0.320, *p* < 0.01). The health behavior change mechanism is significantly negatively correlated with age (*r* = *−*0.279, *p* < 0.01). Physical exercise participation is significantly positively correlated with age (*r* = 0.156*, p* < 0.01).

### 4.5 Hypothesis testing

#### 4.5.1 Model goodness-of-fit test

Table 12 presents the goodness-of-fit test results of the overall model. The X^2^/df (chi-square to degrees of freedom ratio) is 1.523, which falls within the reasonable range of 1–3. The RMSEA (root mean square error of approximation) is 0.033, which is below the threshold of 0.08. Meanwhile, GFI, NFI, RFI, IFI, and CFI values are all greater than 0.9. These results show that the moderated mediation SEM model in this study has a good goodness-of-fit, allowing the hypothesis relationship test of path coefficients to be further analyzed.

**Table 12:** Results of model fit test.

	X^2^/df	GFI	NFI	RFI	IFI	CFI	RMSEA
Model	1.523	0.910	0.917	0.911	0.970	0.970	0.033

#### 4.5.2 Direct effect test

**Figure 3:**
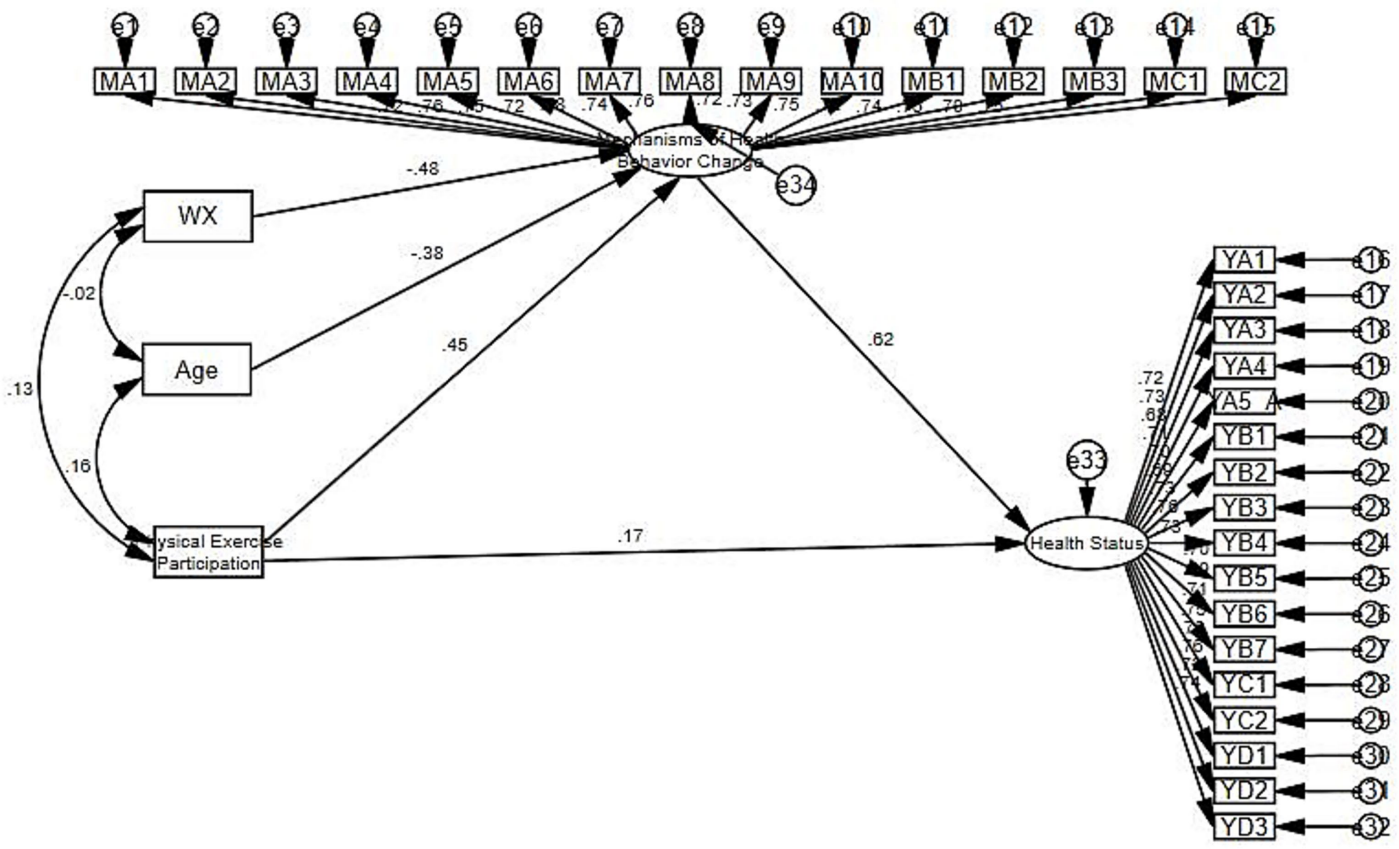
Structural equation model of exercise participation and health status.

The SEM method was used to further verify the research hypotheses, namely to verify the effects of physical exercise participation, age, and the mechanism of health behavior change on health status. Amos 21.0 was used to draw a structural equation model of the effects of physical exercise participation, age, and the mechanism of health behavior change on health status. The sample data from the questionnaire were imported into the model to verify the overall influence mechanism. Finally, a structural equation model containing path coefficients was obtained. The results are presented in Figure 3.

Table 13 presents the results of the relationship tests for each study variable. Physical exercise participation has a significant positive impact on health status (*β* = 0.167, *p* < 0.01). That is, the more an individual participates in physical exercise, the more it can improve their health status. This verifies hypothesis H1. Similarly, physical exercise participation also has a significant positive impact on the health behavior change mechanism (*β* = 0.454, *p* < 0.01). The health behavior change mechanism has a significant positive impact on health status (*β* = 0.622, *p* < 0.01). Age has a significant negative impact on the health behavior change mechanism (*β* = −0.385, *p* < 0.01). Physical exercise can enhance the functions of various organ systems in the body and improve the basal metabolic rate, thereby significantly improving health status. The process of participating in physical exercise can cultivate an individual’s self-discipline and self-management ability, thereby promoting changes in their health behaviors. As individuals age, their physical functions gradually decline and their ability to accept and adapt to new health behaviors decreases, which has a negative impact on the health behavior change mechanism.

**Table 13:** Results of hypothesis testing for coefficients in the structural equation model.

Effect path	Unstd	Estimate	S.E.	C.R.	*p*
Mechanisms of health behavior change ← physical exercise participation	0.003	0.454	0	10.996	***
Mechanisms of health behavior change ← moderating interaction term	−0.257	−0.482	0.022	−11.622	***
Mechanisms of health behavior change ← age	−0.315	−0.385	0.032	−9.721	***
Health status ← physical exercise participation	0.001	0.167	0	4.37	***
Health status ← mechanisms of health behavior change	0.655	0.622	0.058	11.332	***

*p < 0.05; **p < 0.01; ***p < 0.001.

#### 4.5.3 Mediation effect test

We used the bootstrap procedure in Amos 21.0 to further examine the mediating role of the health behavior change mechanism in the relationship between physical exercise participation and individual health status. The confidence interval was set at 95%. If the 95%confidence interval of the indirect effect does not contain 0, this indicates the existence of a mediating effect. The test results are shown in Table 14.

**Table 14:** Results of the mediating effect test.

Effect path	Effect type	Standardized total effects	Bootstrap LLCI	Bootstrap ULCI
Health status ← mechanisms of health behavior change ← physical exercise participation	Total effect	0.904	0.376	0.517
	Direct effect	0.622	0.551	0.685
	Indirect effect	0.282	0.221	0.351

Table 14 shows a standardized indirect effect of 0.282, with a confidence interval of [0.221, 0.351]. Since this interval excludes 0, it confirms that the health behavior change mechanism is a mediator, thereby supporting Hypothesis H2. Additionally, the standardized direct effect is 0.622, with a confidence interval of [0.551, 0.685], which also excludes 0, thereby indicating that the direct path is significant. This suggests that while the health behavior mechanism is a crucial pathway, it may be influenced by other factors, indicating a partial mediating effect. Engaging in physical exercise can increase individuals’ attention to their health, thereby encouraging them to alter detrimental behaviors and activating the mechanism for health behavior change. This shift in health behaviors can lead to the adoption of healthy practices, such as a balanced diet and regular sleep patterns, ultimately enhancing overall health status.

#### 4.5.4 Test of moderating effect

Consistent with the results of the mediation effect test, we used the bootstrap program in Amos 21.0 to test the moderated mediation effect. The results are presented in Table 15. The standardized coefficient of the moderating interaction term physical exercise participation*age is −0.482, and the confidence interval is [−0.571,-0.387], which does not contain 0, indicating that the moderated mediation effect is established. This verifies Hypotheses H3 and H4. That is, increasing age weakens the influence of physical exercise participation on improving health status by enhancing the health behavior change mechanism.

**Table 15:** Test results of the moderated mediation effect.

Effect path	Effect path coefficient	Standardized total effects	Bootstrap LLCI	Bootstrap ULCI
Age regulates health status ← mechanism of health behavior change ← participation in physical exercise	Age	−0.385	−0.458	−0.305
	Physical exercise participation*age	−0.482	−0.571	−0.387

**Figure 4:**
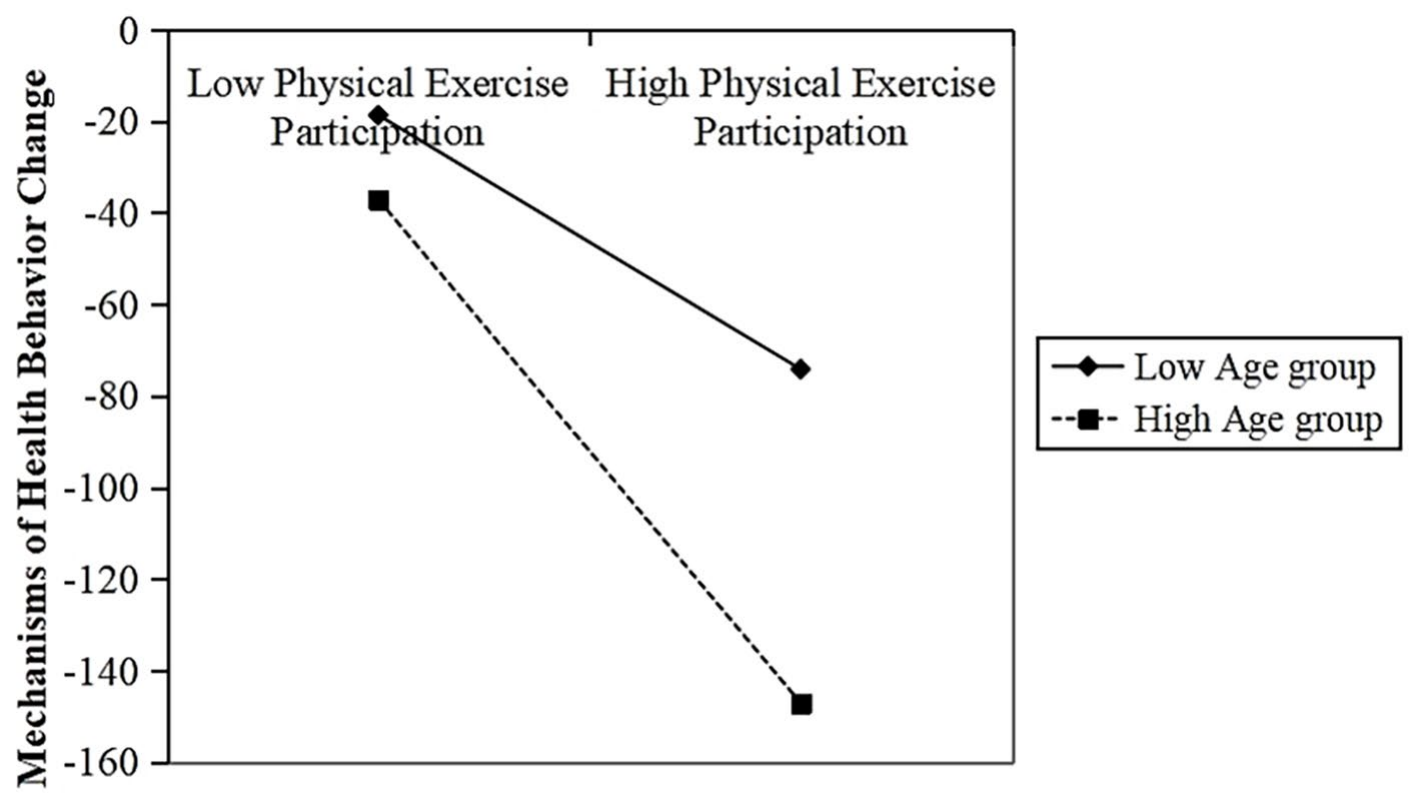
Interaction diagram of age regulation.

Figure 4 depicts an interaction diagram of the moderating effect. The results indicate that the corresponding slope shows a negative inclination in the high-age group, and the degree of inclination is greater, suggesting that age inhibits the mediating effect of health behavior change. That is, age significantly weakens the mediating role of physical exercise participation in influencing physical exercise participation through the mechanism of promoting health behavior change.

Physical functions gradually decline with age, reducing the stimulating effect of physical exercise on the body and weakening the motivation to initiate health behavior changes through exercise. Additionally, ingrained lifestyle habits make it difficult for middle-aged and older adults to accept new behavior patterns, preventing them from fully translating the positive impacts of exercise into comprehensive health behavior changes. Therefore, the older an individual is, the weaker the effect of physical exercise participation will be on improving health status through the mechanism of promoting health behavior changes, exhibiting a moderated mediation effect.

## 5 Discussion

### 5.1 Direct effects of physical exercise in social groups

The impact of physical exercise on the health of middle-aged and older adults has been extensively verified by various studies (60). The present study found that physical exercise in social groups had a significant direct impact on the health status of these age groups (*β* = 0.167, *p* < 0.01), consistent with the findings of previous studies. Healthy aging is crucial for the quality of life of middle-aged and older adults, and there is a strong positive correlation between physical exercise and health (10). It is beneficial for these age groups in reducing medical treatment behaviors and improving health management (61), including improvements in depression or anxiety, physical function, sleep quality, etc. (62). Utilizing the convenient venues of social groups to participate in physical activities (63) can improve these individuals’ perception of their health status (64), and it is also significantly correlated with the active aging level of this demographic (65).

While physical exercise positively influences physical health, the unique social and structural characteristics of the Chinese context significantly shape the relationship between exercise and health. This relationship often manifests as “collective ritualization” (66). Activities such as square dancing and tai chi function not only as physical exercise but also as social interaction events within community public spaces. As sports facilities improve, participants increasingly engage in social communication (67). In this cultural context, collective exercise influences individual health behaviors through the accumulation of social capital, elucidating the complex causal phenomena—primarily driven by direct effects—observed in this study.

Gender differences, the unique features of social activities, and the role of exercise in the context of social group physical activity warrant further exploration. For women, social interaction and spiritual enrichment are often facilitated through communication platforms within social groups, such as tai chi classes. However, aging may create barriers (68) such as challenges in sustaining regular exercise and combating loneliness. In the Chinese context, issues such as the digital divide and competition for space may hinder health outcomes (69). This study examines these dynamics by introducing age as a moderating variable and incorporating several control variables to explore differences. Future research should address multi-dimensional factors, including cultural differences and the general applicability of the findings in both individualistic and collectivistic contexts.

### 5.2 Mediating effect of the mechanism of health behavior change

This study confirmed a partial mediating effect of the mechanism of health behavior change [*β* = 0.282, 95% CI (0.221, 0.35)]. The direct effect (*β* = 0.622) remained significant, however, suggesting that the health benefits of physical exercise are primarily realized through alternative, non-behavior-change pathways. Nonetheless, Hypothesis H2 was validated. This asymmetry challenges the traditional “behavior-centered” model and highlights the causal complexity of exercise-related health benefits (70).

Three potential parallel pathways are proposed to elucidate the source of the direct effect. First, physiological responses, including cardiovascular adaptation, metabolism, and anti-inflammatory effects induced by acute exercise, provide immediate health benefits (71). Second, acute emotional improvements (72) resulting from autonomic nervous system regulation during exercise can directly impact health, bypassing behavioral intentions. Third, elements such as social capital (73), peer support, and social trust (74) may directly influence individual health by fostering a sense of belonging within Chinese social groups (75). This mechanism was not fully captured in the model used in this study.

While the mechanisms underlying health behavior change are complex, consistently maintaining good health habits long-term can significantly enhance the health of middle-aged and older individuals. Some studies have shown that treating the health behaviors of the middle-aged and older as intrinsically motivated can more significantly predict long-term exercise compliance (76), emphasizing the importance of regular and continuous exercise for this group (77). Although many studies have produced inconsistent results regarding the manifestation of the mechanism of health behavior change, the differences among various age groups still require in-depth analysis (78). The present study confirms that, considering multiple factors, a differential analysis of various control variables can reveal the health benefits of physical exercise for the middle-aged and older. A beneficial path for the health impact mechanism can be achieved through multi-directional behavior reorganization.

### 5.3 Moderating effect of age

The influence of age on the relationship between physical exercise and the health of middle-aged and older individuals is a complex, multi-dimensional process. In the context of “collective ritualized” social groups in China, this dynamic involves interactions among physiological, psychological, and social behaviors. This study examines self-psychological efficacy, health responsibility, and behavioral intention as mechanisms for health behavior change. These mechanisms connect participation in physical exercise within social groups to the health status of middle-aged and older individuals. The results show that age significantly moderates the direct relationship between physical exercise participation and health status, as well as the indirect pathways mediated by health behavior change mechanisms, with a standardized coefficient of −0.482 (95%CI:−0.571 to −0.387). Whole-body energy expenditure (79) and muscle mass and function (80) decline with increasing age. Older adults face a series of problems such as difficulties in traveling, a reduction in activity space (81), and age discrimination (82).

Research shows that different age groups exhibit differences in terms of physical activity performance when performing various sports (83). Participating in group sports activities provides an effective way to achieve healthy aging by enhancing the physical activity levels of specific populations, alleviating the decline of cognitive and brain health (84), improving mental health (85), promoting social participation (86), and reducing the risks of various chronic diseases (87). Simple slope analysis further revealed that the motivation for health behavior change through exercise shows a negatively sloped pattern between older and younger age groups. Specifically, the motivation for health behavior change is weaker in the older group compared to the younger one, which may be attributed to the cognitive biases in health behavior habits among the older. Future research should continue to explore the factors underlying this mechanism and pay particular attention to age-specific and culturally sensitive group sports intervention measures to maximize health benefits.

### 5.4 Research limitations and future directions

While this study confirmed the relationships among group physical exercise, health behavior change mechanisms, age, and health status in the middle-aged and older population in China, several limitations warrant consideration. First, the cross-sectional design precludes causal inferences. While reverse causality is plausible, the health behavior change mechanism in a collective context involves complex causal relationships influenced by multiple factors. Future research should employ longitudinal or experimental designs to capture the dynamic characteristics of these mechanisms. Second, the study’s high proportion of female participants may limit the generalizability of the findings to male individuals. This imbalance likely stems from typical participation patterns among Chinese women in social groups such as Taijiquan and square dancing. Future studies should prioritize gender-balanced sampling to examine gender specificity. Third, the sample was primarily drawn from Sichuan Province, which has a homogeneous cultural background, potentially leading to cultural generalization of the findings. Future research should broaden the sampling area and emphasize the impact of cultural diversity. Fourth, age was used as a moderating variable, but due to sample size constraints, participants aged 76 and older were combined with the 66–75 age group. This classification may obscure characteristics of the older population and mask non-linear effects. Future research should expand the sample size of the oldest group and treat age as a continuous variable to optimize the model. Fifth, while the PASE scale has been validated in multiple studies, its application within the “social group” context in this study creates a non-traditional combination of scale items as the independent variable, potentially influenced by common expectation bias in social organization environments. Using objective data can enhance construct validity.

## 6 Conclusion

This study developed a moderated mediation model to reinterpret the relationship between physical exercise and health within social environments. The findings indicate that, in the Chinese context, group physical exercise both directly influences the health status of middle-aged and older individuals and indirectly affects it through a health behavior change mechanism. This mechanism comprises self-efficacy, health responsibility, and behavioral intention. Notably, the effect diminishes with age, with a more pronounced decline observed in older age groups than in younger ones. This research integrates perspectives from social ecology and life-cycle theories to enhance our understanding of how the health behavior change mechanism in China promotes the health of middle-aged and older individuals across various life stages. It also provides a precise framework for healthy aging initiatives.

## Data Availability

The original contributions presented in the study are included in the article/supplementary material, further inquiries can be directed to the corresponding author.
